# Expression of human *ARGONAUTE 2* inhibits endogenous microRNA activity in *Arabidopsis*

**DOI:** 10.3389/fpls.2013.00096

**Published:** 2013-04-16

**Authors:** Ira Deveson, Junyan Li, Anthony A. Millar

**Affiliations:** Plant Sciences Division, Research School of Biology, Australian National UniversityCanberra, ACT, Australia

**Keywords:** HsAGO2, AtAGO1, *ago1–27*, microRNA, Arabidopsis

## Abstract

Plant and animal microRNA (miRNA) pathways share many analogous components, the ARGONAUTE (AGO) proteins being foremost among them. We sought to ascertain the degree of functional conservation shared by *Homo sapiens ARGONAUTE 2* (*HsAGO2*) and *Arabidopsis thaliana ARGONAUTE 1* (*AtAGO1*), which are the predominant AGO family members involved with miRNA activity in their respective species. Transgenic *Arabidopsis* plants expressing *HsAGO2* were indistinguishable from counterparts over-expressing *AtAGO1*, each group exhibiting the morphological and molecular hallmarks of miRNA-pathway loss-of-function alleles. However, unlike *AtAGO1, HsAGO2* was unable to rescue the *ago1–27* allele. We conclude that, despite the evolutionary gulf between them, *HsAGO2* is likely capable of interacting with some component/s of the *Arabidopsis* miRNA pathway, thereby perturbing its operation, although differences have arisen such that HsAGO2 alone is insufficient to confer efficient silencing of miRNA targets *in planta*.

## Introduction

MicroRNAs (miRNAs) are endogenous small RNAs (sRNAs) that direct the sequence-specific silencing of mRNA transcripts, providing a critical layer of gene regulation in both plants and animals (Pasquinelli, 2012). Much of the molecular machinery that oversees miRNA biogenesis and activity is shared between these kingdoms and, hence, must have arisen prior to their divergence, though it is unclear whether a basal miRNA pathway was already in operation at this time (Axtell et al., 2011). From this shared origin, the plant and animal miRNA pathways have followed divergent evolutionary courses and there now exists characteristic distinctions in the manner of their operation. Briefly, plant miRNAs generally silence only a small handful of targets, requiring near-perfect complementarity for their recognition, and do so via a combination of transcript cleavage and a non-cleavage mechanism/s (Brodersen and Voinnet, 2009). In contrast, animal miRNAs regulate target transcripts to which they are only partially complementary, generally recognizing many targets and silencing these via a non-cleavage mechanism/s (Axtell et al., 2011; Huntzinger and Izaurralde, 2011). The question of how the apparently similar set of components in the miRNA pathways of plants and animals has been modified so as to generate these functional differences is keenly relevant to our understanding of eukaryotic gene regulation.

The best example of shared componentry between the systems, an ARGONAUTE (AGO) protein is required, without known exception, for miRNA-mediated gene silencing (Mallory and Vaucheret, 2010). Considered the core component of the miRNA induced silencing complex (miRISC), AGO's involvement in miRNA-mediated silencing appears threefold. First, it facilitates the interaction of a miRNA with its intended targets (Nakanishi et al., 2012; Schirle and Macrae, 2012). Second, when sufficient complementarity exists, it provides the catalytic activity for cleavage of the target mRNA (Liu et al., 2004; Meister et al., 2004; Baumberger and Baulcombe, 2005; Rivas et al., 2005). Finally, it orchestrates the silencing of un-cleaved targets by recruiting the factors responsible for their translational repression and accelerated degradation (Huntzinger and Izaurralde, 2011).

Plants and animals each exhibit a diversity of AGO proteins. There are, for example, 10 members in *Arabidopsis*, which are distinguished by the class of sRNAs that they associate with and by their patterns of expression (Vaucheret, 2008; Mallory and Vaucheret, 2010). Of these, *Arabidopsis thaliana AGO1* (*AtAGO1*) has the most prominent involvement in miRNA-mediated gene silencing. Emphasizing the importance of its role, *AtAGO1* homeostasis must be delicately maintained by a feedback mechanism in which miR168 directs the *AtAGO1*-dependent silencing of *AtAGO1* expression. When this circuit is disturbed via the mutation of *AtAGO1*'s miR168 target site, the over-accumulation of AtAGO1 protein leads—somewhat counter intuitively—to a general perturbation of miRNA-mediated regulation in the plant, manifesting in pleiotropic defects that resemble those apparent in a range of miRNA-pathway loss-of-function alleles (Morel et al., 2002; Vaucheret et al., 2004; Vazquez et al., 2004).

Of four mammalian AGOs, *Homo sapiens AGO2* (*HsAGO2*) has the best-characterized role in the miRNA pathway. Although HsAGO2 and AtAGO1 share only 43% amino acid identity (Poulsen et al., 2013), they appear similar in function. Both preferentially associate with sRNAs bearing a 5′U (Mi et al., 2008; Frank et al., 2010) and may catalyze the cleavage of complementary mRNA transcripts *in vitro* without the participation of any other component (Liu et al., 2004; Meister et al., 2004; Baumberger and Baulcombe, 2005; Rivas et al., 2005). Moreover, they interact and operate in conjunction with proteins of analogous structure and function. For example, HsAGO2 facilitates the non-cleavage silencing of targets (Pillai et al., 2004) in a process dependent on the GW182 family proteins (Takimoto et al., 2009; Zipprich et al., 2009), while AtAGO1 (Brodersen et al., 2008; Lanet et al., 2009) and an *Arabidopsis* GW protein SUO (Yang et al., 2012) each have a demonstrated involvement in miRNA-mediated translational repression. The plant de-capping factor VARCIOSE (VCS) (Brodersen et al., 2008) and its animal ortholog Ge-1 (Eulalio et al., 2007) have also been implicated in translational repression. Further to this, the loading of miRNAs into AtAGO1 and HsAGO2 is supported by chaperones that are conserved between the two kingdoms (Smith et al., 2009; Iki et al., 2010, 2012; Iwasaki et al., 2010; Earley and Poethig, 2011).

The array of similarities just outlined indicates that, despite the evolutionary distance between them, *AtAGO1* and *HsAGO2* share a remarkable degree of functional conservation. Indeed, in their recent report, Poulsen et al. (2013) integrated information from mutational studies of *AtAGO1* with structural studies of HsAGO2 to make general inferences about the mechanics of AGO activity, the underlying assumption being that these two proteins are highly similar in form and function. By expressing *HsAGO2* in *Arabidopsis*, we sought to determine whether they might be so similar that HsAGO2 could function as a component of the plant miRNA pathway or whether distinctions that have accumulated during their parallel evolution would become apparent when expressed in an identical cellular context, perhaps providing insight into the characteristic operational differences of the plant and animal miRNA pathways.

## Materials and methods

### Plant materials and growth conditions

Seeds were surface sterilized by exposure (3–6 h) to the chlorine gas generated by mixing 100 mL of sodium hypochlorite with 3 mL of concentrated hydrochloric acid in a sealed desiccator jar. Seeds were sown on soil (Debco Plugger mixed with Osmocote Extra Mini fertilizer at 3.5 g/L), stratified for 48 h at 4°C and grown under “long day” conditions (16 h light/8 h dark, 150 μmol/m^2^/s, 22°C).

### Generating *35s:HsAGO2, 35s:AtAGO1* and *35s:4m-AGO1* transgenic plants

*HsAGO2* and *AtAGO1* cDNAs were obtained from Sino Biological and the *Arabidopsis* Biological Resource Centre respectively. Via standard Gateway cloning procedures, each was placed into the pMDC32 destination vector, which contains a double 35S promoter for constitutive expression (Curtis and Grossniklaus, 2003). For the *35S:4mAGO1* construct, four silent mutations introducing mismatches to the miR168 target site in *AtAGO1*, as per (Vaucheret et al., 2004), were generated via a site directed mutagenesis strategy based on that of (Liu and Naismith, 2008). Constructs were transformed into *Agrobacterium tumefaciens* and then transformed into *Arabidopsis* using the “floral dip” method (Clough and Bent, 1998).

### RNA extraction and cDNA synthesis

Total RNA was extracted from whole rosettes of plants at different growth stages, using TRIzol (Invitrogen) with the following modifications made to the manufactures protocol: (1) approximately 500 mg of plant material was used with 1 mL of Trizol reagent for each extraction; (2) homogenization of tissues was achieved using a mortar and pestle; (3) the chloroform extraction step was repeated twice; (4) precipitation of RNA was carried out overnight at −20°C to maximize the recovery of sRNAs. 30–50 μg of RNA from each sample was treated with RQ1 RNase-Free DNase (Promega) in separate 100 μL reactions, according to the manufacturer's protocol, with the addition of RNaseOut Recombinant RNase Inhibitor (Invitrogen) (1 μL/10 μg RNA). Treated RNA was then purified using Qiagen RNAeasy cleanup kit according to the manufacturer's protocol. cDNA synthesis was carried out using SuperScript III Reverse Transcriptase (Invitrogen) and an oligo dT primer according to manufacturer's protocol. For each sample, 250 ng −5 μg of purified RNA was used in separate 20 μL reactions. These were subsequently diluted in 980 μL nuclease free distilled water before qRT-PCR analysis.

### Quantitative real-time PCR (qRT-PCR)

For qRT-PCR, 9.2 μL of each cDNA sample was added to 10 μL of SensiFAST SYBR No-ROX mix (Bioline) with 0.8 μL of forward and reverse primers (10 μmol each). For the measurement of un-cleaved mRNA levels of miRNA target genes, qRT-PCR primers were designed so their amplicon would span the target site for their associated miRNAs, meaning that cleaved transcripts would not contribute to the measured abundance. qRT-PCR reactions were carried out on a Rotor-Gene 2000 real time PCR machine (Qiagen) in triplicate. The “housekeeper” *CYCLOPHILIN* (At2g29960) was used to normalize mRNA levels of each gene using the comparative quantitation program in the Rotor-Gene 6 software package provided by Qiagen and average values were calculated from triplicate measurements.

### qRT-PCR assays for mature miRNAs

Customized Taqman sRNA assays (Applied Biosystem) were used to quantitate mature miRNAs and amiRNAs according to the manufacturer's protocol, apart from the following modifications: (1) for each RNA sample, the retro-transcription was multiplexed with looped-RT primers for the miRNA/amiRNA of interest and the small RNA *sno101*; (2) the cDNA synthesized (15 μL) was diluted with 86.4 μL nuclease free distilled water and 9 μL of this cDNA solution was used in each qRT-PCR reaction, along with 10 μL SensiFAST Probe NO-ROX mix (Bioline) and 1 μL Taqman probe (Applied Biosystems). qRT-PCR reactions were carried out on a Corbett Rotor-Gene 2000 real time PCR machine (Corbett) in triplicate as above. The abundance of each mature miRNA/amiRNA was normalized to *sno101* using the comparative quantitation analysis program from Rotor-Gene 6 software (Corbett) and average values were calculated from triplicate measurements.

## Results

### Expression of *HsAGO2* in *Arabidopsis* results in pleiotropic developmental defects

**Figure 1:**
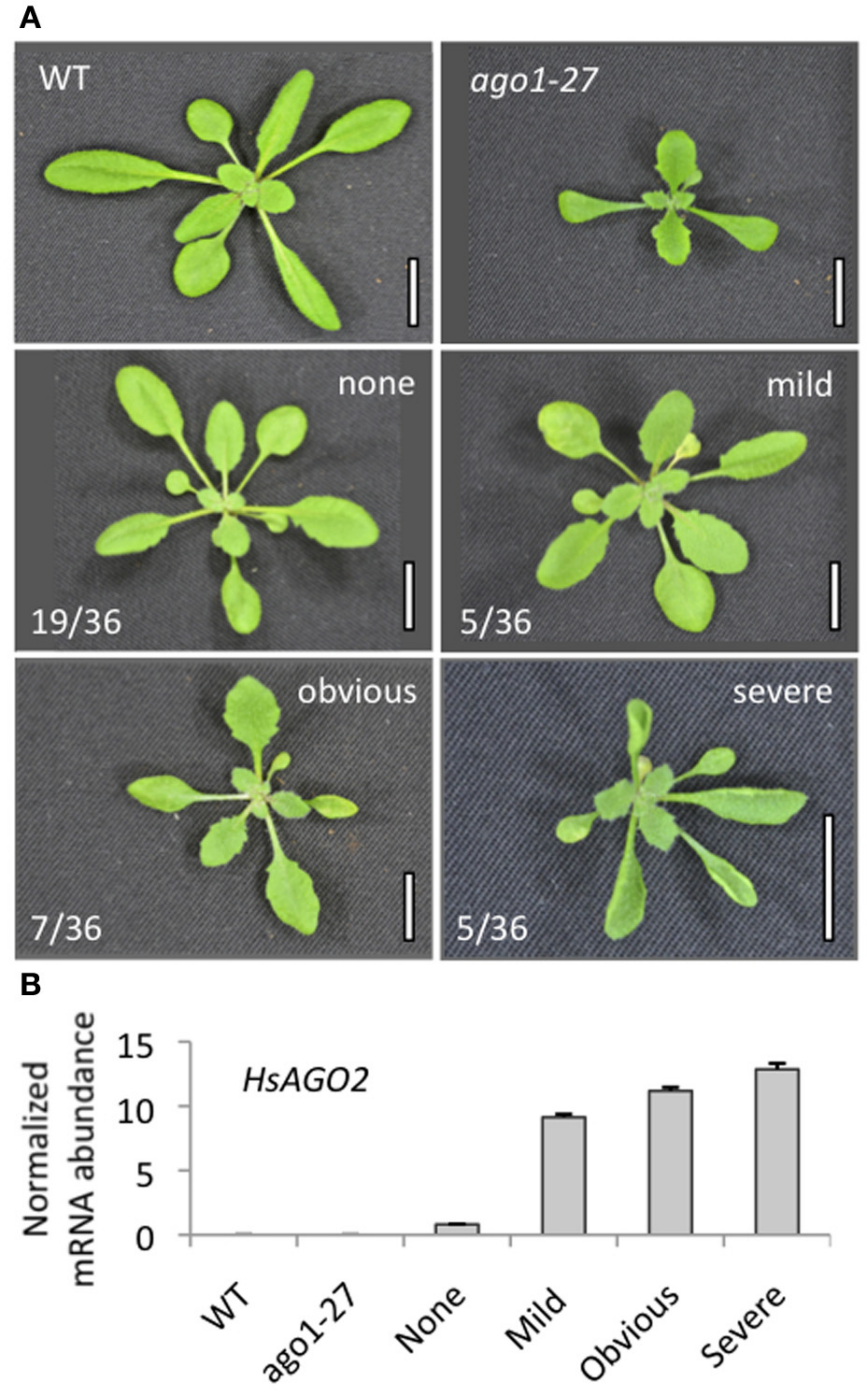
**Expression of HsAGO2 generates morphological defects in Arabidopsis. (A)** 22-day old primary transformants for the *35S:HsAGO2* construct were categorized based on the apparent severity of their morphological phenotypes. Increased leaf serration distinguished “mild” phenotypes from wild type (WT), “obvious” phenotypes were characterized by broadened leaves, serration, accelerated senescence and some upward leaf-curl, while phenotypes considered “severe” were distinguished by strong upward leaf-curl in addition. Scale bars represent 10 mm. **(B)** The abundances of *HsAGO2* mRNA was measured in total RNA from sample pools composed of 4–8 transformants, 22-days old, from each morphological category. Wild type (WT) and *ago1–27* plants, grown in parallel, were included as controls. All measurements are normalized to *CYCLOPHILIN* mRNA. Data is averaged from three technical cDNA replicates, each of which comprised triplicate measurements, and error bars depict standard error of the mean.

A cDNA for *HsAGO2* was placed under the control of a double 35S promoter and the resulting binary vector (*35S:HsAGO2*) was transformed into wild type *Arabidopsis*. Roughly half (17/35) of all *35S:HsAGO2* primary transformants displayed morphological abnormalities of varying severity. At its mildest, the *35S:HsAGO2* phenotype was characterized by increased leaf-serration relative to wild type. Reduced rosette size, accelerated senescence and flat, broad leaves were additional features in more severely affected transformants and, in several, upward leaf-curl was also apparent (Figure 1A). Plants were grouped according to the perceived severity of their abnormalities and these assignments were well correlated with the abundance of *HsAGO2* mRNA recorded in each set (Figure 1B). Hence, the expression of *HsAGO2* is able to generate pleiotropic developmental defects in *Arabidopsis.*

### *HsAGO2* expression and *AtAGO1* over-expression generate indistinguishable morphological phenotypes in *Arabidopsis*

**Figure 2:**
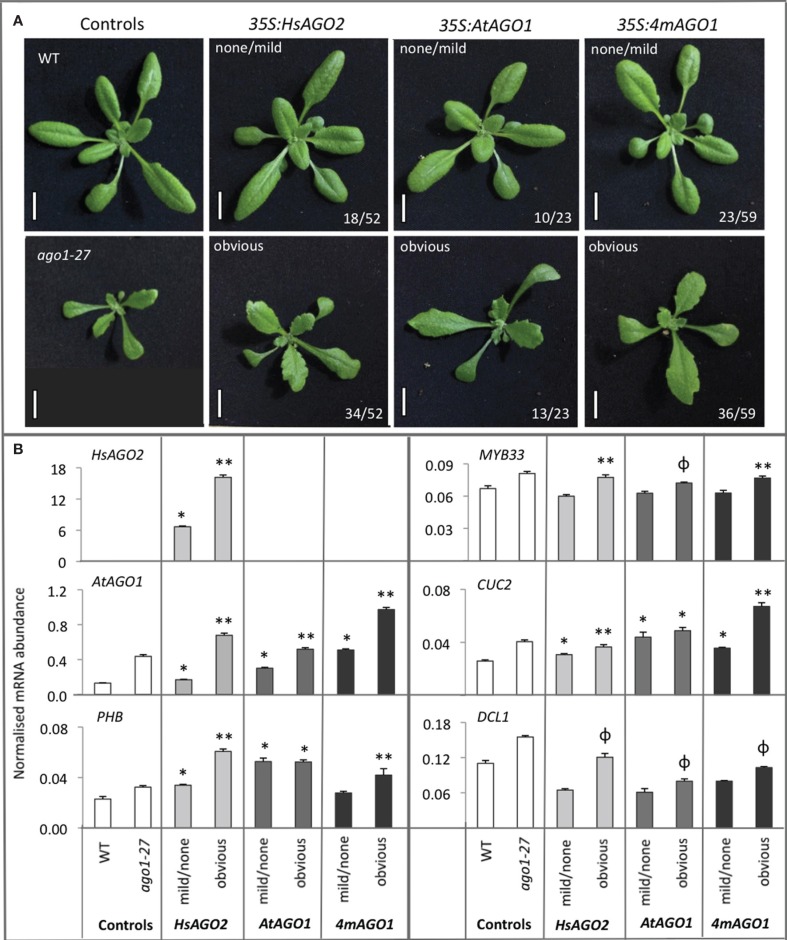
**Overexpression of HsAGO2 or AtAGO1 results in similar morphological and molecular phenotypes. (A)** 24-day old primary transformants for the *35S:HsAGO2*, *35S:AtAGO1*, and *35S:4mAGO1* constructs, grown in parallel, were categorized as exhibiting an obvious abnormal morphological phenotype, characterized by broad, flattened, serrated leaves, or no/mild abnormal phenotype. Wild type (WT) and *ago1–27* plants were grown in parallel as comparators. Scale bars represent 10 mm. **(B)** The abundances of *HsAGO2* mRNA and un-cleaved mRNA for *AtAGO1, PHB, MYB33*, *CUC2*, and *DCL1* were measured in total RNA from sample pools composed of 4–8 transformants, 24-days old, from each morphological category for each construct. Wild type (WT) and *ago1–27* plants, grown in parallel, were included as controls. Measurements of “un-cleaved” mRNA are obtained by using a qRT-PCR amplicon spanning the cleavage site for each transcript, such that cleaved mRNA does not contribute to the recorded abundance. All measurements are normalized to *CYCLOPHILIN* mRNA. Data is averaged from two technical cDNA replicates, each of which comprised triplicate measurements, and error bars depict standard error of the mean. Values marked with ^*^ are significantly larger (*P* < 0.05) than their corresponding measurements in WT samples, those with ^**^ are significantly larger (*P* < 0.05) than both WT and their corresponding none/mild sample, whilst those marked with ϕ are significantly larger (*P* < 0.05) than their corresponding none/mild sample but not WT.

The serration and upward leaf-curl seen in *35S:HsAGO2* transgenic plants are features observed in a number of previously described miRNA-pathway loss-of-function alleles (Morel et al., 2002; Vaucheret et al., 2004; Vazquez et al., 2004), hinting that endogenous miRNA activity might be perturbed by *HsAGO2* expression. Since over-expression of endogenous *AtAGO1*, elicited by the mutation of its miR168 target site, leads to a miRNA-pathway loss-of-function effect (Vaucheret et al., 2004, 2006), the excessive expression of *HsAGO2*, unregulated by any homeostatic mechanism, might perturb miRNA activity in a similar fashion. To test this, we wished to directly compare *35S:HsAGO2* plants to transgenic plants over-expressing *AtAGO1*. To generate these, a cDNA for *AtAGO1* was placed under a double 35S promoter (*35S:AtAGO1*) and, in a separate construct (35S:*4mAGO1*), four silent mutations identical to those described by Vaucheret et al. (2004) were created, introducing four mismatches to *AtAGO1*'*s* miR168 target site, which should render it resistant to miR168-mediated regulation. Both constructs were transformed into wild type plants and their primary transformants were grown alongside *35S:HsAGO2* plants. Populations of transformants for all three constructs were morphologically indistinguishable, each eliciting phenotypes characterized by serration and broad, flattened leaves (Figure 2A). These abnormal phenotypes were associated with elevated *AtAGO1* mRNA levels (Figure 2B), ruling out co-suppression of the endogenous gene as an alternative explanation. The similarity of plants expressing *HsAGO2* to plants over-expressing *AtAGO1* is consistent with the possibility that *HsAGO2* expression perturbs endogenous miRNA activity and suggests that the HsAGO2 protein behaves similarly to AtAGO1 in an over-expression context.

### *HsAGO2* plants exhibit molecular characteristics of miRNA-pathway loss-of-function alleles

Seeking further evidence that *HsAGO2* expression perturbs endogenous miRNA activity, the abundance of un-cleaved mRNA for each of five miRNA targets was measured in *HsAGO2* transformants that displayed obvious or mild/no morphological abnormalities. The same analysis was performed for *35S:AtAGO1* and *35S:4mAGO1* transformants, with wild type plants and *ago1–27*, a partial loss-of-function allele of *AtAGO1* (Morel et al., 2002), serving as controls.

The five miRNA targets examined, *AtAGO1* (miR168), *DCL1* (miR162), *PHABULOSA* (*PHB*) (miR165/166), *MYB33* (miR159), and *CUP-SHAPED COTYLEDONS 2* (*CUC2*) (miR164), all showed elevated un-cleaved mRNA abundances in *ago1–27* plants relative to wild type (Figure 2B), consistent with what has previously been reported (Morel et al., 2002). *HsAGO2* plants displaying obvious aberrant phenotypes also exhibited greater mRNA accumulation than transformants showing no/mild aberrant phenotypes or wild type comparators for *AtAGO1, PHB, MYB33*, and *CUC2* (*P* < 0.05) (Figure 2B). The same was generally true for *35S:AtAGO1* and *35S:4mAGO1* transformants and it could not be said that, across the board, any one of the three constructs generated a more severe molecular phenotype than the other two (Figure 2B). For each construct, *DCL1* levels were not significantly increased relative to wild type but were higher in transformants that displayed an obvious aberrant phenotype than those that did not (*P* < 0.05) (Figure 2B). Thus, the exhibition of morphological defects among transformants of all three constructs corresponded with a general increase in the mRNA abundance of miRNA targets, implying a perturbation of miRNA activity in each.

**Figure 3:**
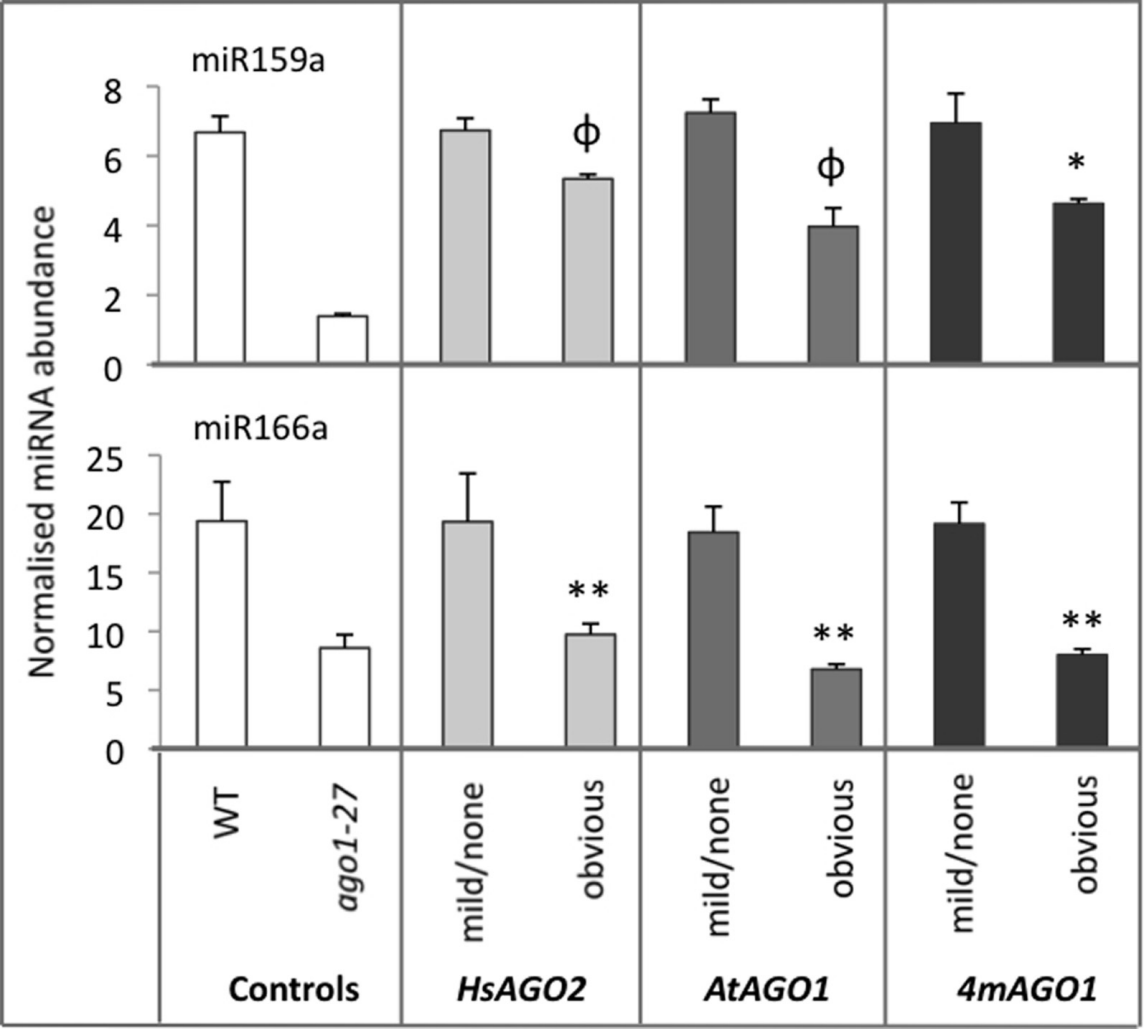
**miRNA abundances decrease in AGO overexpressing plants.** The abundances of miR159a and miR166 were measured in total RNA from sample pools composed of 4–8 transformants, 24-days old, from each morphological category for each construct. Wild type (WT) and *ago1–27* plants, grown in parallel, were included as controls. miRNA levels are normalized to the small RNA sno101. Data is averaged from two technical cDNA replicates, each of which comprised triplicate measurements, and error bars depict standard error of the mean. Values marked with ^*^ are significantly smaller (*P* < 0.05) than corresponding measurement in WT samples, those with ^**^ are significantly smaller (*P* < 0.05) than both WT and their corresponding none/mild sample, whilst those marked with φ are significantly smaller (*P* < 0.05) than their corresponding none/mild sample but not WT.

The apparent perturbation of miRNA activity in plants expressing *HsAGO2* at high levels seems to imply an interaction between the HsAGO2 protein and some component/s of the endogenous miRNA pathway, the most obvious candidate being miRNA molecules themselves. Hence, mature miRNA levels for two highly abundant miRNAs, miR159a and miR166, were measured in *35S:HsAGO2* transformants from the same morphological categories as above, with *35S:AtAGO1* and *35S:4mAGO1* transformants, wild type and *ago1–27* again included for comparison. Transformants of all three constructs displaying no aberrant phenotype showed miRNA accumulation roughly equivalent to wild type (Figure 3). MiR159a and miR166 levels were decreased in transformants displaying an obvious aberrant phenotype, though these decreases were not always found to be statistically significant compared to wild type (*P* < 0.05) (Figure 3). Since miRNA levels were also lower in *ago1–27* than in wild type (Figure 3), this result is consistent with the notion that the three constructs behave as miRNA-pathway loss-of-function alleles. The decreased accumulation of mature miRNAs could not be explained by changes at the transcriptional level, since parallel decreases in pri-miRNA abundances were not observed (data not included).

In sum, *35S:HsAGO2* transformants and *AtAGO1* over-expressers are morphologically indistinguishable, display similarly elevated mRNA abundances for five miRNA targets and comparable reductions in the accumulation of two mature miRNAs. Collectively, these findings strongly argue that HsAGO2 inhibits endogenous miRNA activity and behaves in a similar fashion to its plant counterpart, at least in an over-expression context.

### *35s:HsAGO2* is unable to rescue the *ago1–27* allele

**Figure 4:**
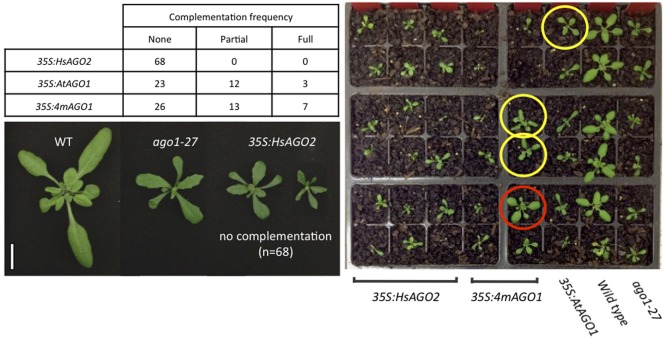
***HsAGO2* cannot complement the *ago1–27* mutation.** 22-day old transformants of *35S:HsAGO2*, *35S:AtAGO1*, and *35S:4mAGO1* in the *ago1–27* background were categorized based on their morphologies as being non-complemented (resembling *ago1–27)*, partially complemented (intermediate phenotype) or fully complemented (resembling wild type). The left image shows representative non-complemented transformants for the *35S:HsAGO2* construct, alongside wild type (WT) and *ago1–27* controls. Scale bar represents 10 mm. The right image shows a representative group of transformants for each construct, alongside WT and *ago1–27*, with plants that were scored as being partially (yellow) or fully complemented (red) highlighted.

That *HsAGO2* expression generates a miRNA-pathway loss-of-function phenotype does not necessarily imply that the protein lacks functionality in the plant cell, since the over-expression of *AtAGO1* similarly inhibits endogenous miRNA activity. With the anticipation that a functional HsAGO2 protein might alleviate an *ago1* loss-of-function allele, the *35S:HsAGO2* construct was transformed into *ago1–27*, as were *35S:AtAGO1* and *35S:4mAGO1*. Both *AtAGO1* constructs were able to complement the *ago1–27* morphological phenotype, yielding transformants resembling wild type plants with similar frequencies. Full or partial complementation was observed in 15/38 transformants of *35S:AtAGO1* and 20/46 for *35S:4mAGO1* (Figure 4). This implies that, although both constructs can inhibit miRNA activity in a wild type background, they are also able to fully or partially restore AGO1 activity in the *ago1–27* mutant background. By contrast, none of the 68 transformants for *35S:HsAGO2* showed even partial phenotypic complementation, with the majority exhibiting an even more severely aberrant phenotype than *ago1–27* (Figure 4). This result argues that, whilst HsAGO2 behaves just like AtAGO1 in an over-expression context, the human protein, unlike its plant counterpart, is seemingly incapable of facilitating the efficient silencing of miRNA targets in *Arabidopsis*.

## Discussion

In both plants and animals, an AGO protein is required for miRNA-mediated gene silencing. The best-studied AGO proteins of each kingdom, AtAGO1 and HsAGO2 are believed to share a high degree of functional conservation. Seeking to determine the full extent of their similarity, we constitutively expressed *HsAGO2* in *Arabidopsis*. *35S:HsAGO2* transformants displayed abnormal phenotypes indistinguishable from those of transgenic plants over-expressing *AtAGO1*, with both groups resembling miRNA-pathway loss-of-function alleles at the morphological and molecular levels and exhibiting similar decreases in the abundances of mature miRNAs. We are aware of no other attempt to express a component of the animal miRNA pathway *in planta*, or vice versa, and our results indicate that, despite the evolutionary gulf between the two systems, the HsAGO2 protein retains the ability to interact with some component/s of the plant miRNA pathway and behaves similarly to AtAGO1 in an over-expression context.

Whilst *HsAGO2* behaves just like *AtAGO1* in an over-expression context, it was unable to rescue the *ago1–27* allele, indicating that it is insufficient for the efficient silencing of miRNA targets in *Arabidopsis*. This is unsurprising, given that even the closest homologue of *AtAGO1*, *AtAGO10*—with which it shares 75% amino acid identity compared to just 43% for *HsAGO2* (Poulsen et al., 2013)—expressed from *AtAGO1*'s promoter, is unable to rescue the same allele (Zhu et al., 2011). In fact, ectopic expression of *AtAGO10* from the *AtAGO1* promoter yielded upward leaf-curl and exacerbated the *ago1–27* allele (Zhu et al., 2011), just as we observed for the *35S:HsAGO2* construct. While AtAGO10 associates with miRNAs and is capable of cleaving target transcripts *in vitro*, it is not an efficient gene-silencer *in vivo* and instead attenuates AtAGO1-associated miRNA activity by sequestering miRNAs, having a particular preference for miR165/166 (Zhu et al., 2011). The HsAGO2 protein is also sufficient for sRNA-directed mRNA cleavage *in vitro* (Liu et al., 2004; Meister et al., 2004; Rivas et al., 2005) but apparently fails to efficiently silence miRNA targets *in planta*. It seems therefore that a basal cleavage activity, demonstrable *in vitro*, is not sufficient for proper miRNA-mediated gene silencing *in planta.* Consequently, we suggest that the AtAGO1 protein is distinguished by some specialization, perhaps allowing it to interact with an unknown factor/s that is additionally required for efficient silencing, which is essential for its function. The requirement for an additional factor/s could explain the somewhat counterintuitive observation that *AtAGO1* over-expression generates a miRNA-pathway loss-of-function effect; an excess of AtAGO1 protein might titrate miRNAs and other necessary components of the miRISC into separate, incomplete complexes, each unable to facilitate miRNA-guided gene silencing, thereby inhibiting endogenous miRNA activity. Such an explanation was proposed by Vaucheret et al. (2004), who first reported a perturbation of miRNA activity by *AtAGO1* over-expression. Here it must be noted that Vaucheret et al. (2006) reported modest increases in the accumulation of mature miR159a and miR166 in transgenic plants over-expressing *AtAGO1*, the opposite result to what we observed. The foundation of this contradiction might lie in the fact that the two studies employed different protocols for the extraction of total RNA from plant tissues, although this explanation cannot be substantiated here. That both the *AtAGO1* and the *4mAGO1* transgenes were, in the present study, expressed from a constitutive double 35S promoter in, as opposed to the endogenous promoter of *AtAGO1* (Vaucheret et al., 2006), is another point of difference between the two approaches, and may have contributed to their divergent outcomes.

The miRNA pathways of plants and animals utilize similar molecular machinery but exhibit characteristic distinctions in the manner of their operation, presumably the manifestation of componential modifications that have accumulated throughout their parallel evolutionary histories. The most important of these is the differential requirements for target recognition in each, animal miRNAs commonly regulating targets that would be ignored in plants for their insufficient complementarity (Axtell et al., 2011). Given the intimacy of their involvement in miRNA-mediated gene silencing, one could reasonably speculate that some intrinsic difference in the AGO proteins of plants and animals might underpin their differential target specificities. Because HsAGO2 was apparently unable to efficiently silence miRNA targets *in planta*, we were unable to address the possibility that an animal and a plant AGO might regulate distinct sets of targets when expressed in an identical cellular context. However, it was recently reported that cleavage-impaired *AtAGO1* mutants, which are defective in gene silencing activity, successfully co-precipitate with both miRNAs and mRNAs, suggesting that their ability to recognize and bind to target transcripts remains intact (Carbonell et al., 2012). Thus, an avenue for future work will be to attempt to elucidate the target profile of HsAGO2 *in planta* via co-precipitation experiments, potentially revealing differential specificities for an animal and plant AGO.

### Conflict of interest statement

The authors declare that the research was conducted in the absence of any commercial or financial relationships that could be construed as a potential conflict of interest.
